# Effects of Fused Silica Addition on Thermal Expansion, Density, and Hardness of Alumix-231 Based Composites

**DOI:** 10.3390/ma15103476

**Published:** 2022-05-12

**Authors:** Luciano M. Rodrigues, Bojan A. Marinkovic

**Affiliations:** Department of Chemical and Materials Engineering, Pontifícia Universidade Católica do Rio de Janeiro (PUC-Rio), Rio de Janeiro 22453-900, RJ, Brazil; luciano_rodrigues@id.uff.br

**Keywords:** powder metallurgy, liquid phase sintering, ceramic filler, coefficient of thermal expansion

## Abstract

Fused silica is a ceramic with promising applications as a filler in composites due to its near-zero thermal expansion. Substitution of heavy cast iron with Al-based light alloys is of utmost importance for the automotive industry. However, the high thermal expansion of Al alloys is an obstacle to their use in some applications. As such, ceramic fillers are natural candidates for tuning thermal expansion of Al-based matrices, due to their inherently moderate or low thermal expansion. Alumix-231 is a new promising alloy, and fused silica has never been used before to lower its thermal expansion. Composites with the addition of 5 to 20 vol.% of fused silica were developed through powder metallurgy, and the best results in terms of reduction of thermal expansion were reached after liquid phase sintering at 565 °C. Coefficients of thermal expansion as low as 13.70 and 12.73 × 10^−6^ °C^−1^ (between 25 and 400 °C) were reached for the addition of 15 and 20 vol.% of fused silica, a reduction of 29.9% and 34.8%, respectively, in comparison to neat Alumix-231. In addition, the density and hardness of these composites were not significantly affected, since they suffered only a small decrease, no higher than 6% and 5%, respectively. As such, the obtained results showed that Alumix-231/fused silica composites are promising materials for automotive applications.

## 1. Introduction

Over the decades, the transition from iron-based heavy alloys to lighter alloys has continued to gain more attention in the automotive and aerospace industries, due to the growing demand for weight reduction, improved fuel economy, and, therefore, less pollutant vehicles. To be considered for such purposes, light alloys must have sufficiently high mechanical properties (i.e., hardness and tensile strength) and, in some cases, well-controlled coefficients of thermal expansion [1,2,3,4,5,6]. The need for new materials capable of meeting increasingly stringent requirements has led to the development—in the last two decades—of low-weight metal matrix composites (MMCs). Aluminum and its alloys are among the main matrices in such MMCs, being light and high-performance materials with potential and effective applications in the automotive, aerospace, electronics, and military industries [7,8,9,10]. Powder metallurgy (P/M) is considered a suitable technique in producing these composites, mainly due to the lower processing temperatures compared to casting techniques, and the uniform distribution of reinforcement particles within the matrix [11].

One of the more recent light Al alloys—with a density as low as 2.634 g cm^−3^ [12]—is Alumix-231, with a nominal chemical composition of Al-15Si-2.5Cu-0.5Mg. This alloy consists of a mixture of neat aluminum powder and a hypereutectic master alloy (Al-28Si-5Cu-1Mg wt.%). When compared to other Al alloy systems, the Al-Si family is an excellent competitor since it offers lower densities and coefficients of thermal expansion (CTE) [13], making it potentially suitable for different types of precision components in the automotive industry [14]. Although Alumix-231 was the first commercial system based on Al-Si to undergo P/M processing, the amount of data available in the literature on this alloy is still limited [5,9,12,15,16,17,18].

Since P/M is a promising technology capable of producing new lightweight components, Al-Si-based alloys processed in this way are considered as potential candidates for replacing conventional cast and forged components due to the low material waste, lower processing temperatures and costs, and capability of production of forms with complex geometry and—on a high scale of production—close to liquid-shaped bodies, such as sprockets, cylinder liners, connecting rods, pistons, etc. [5,9,12,13,15,16,17,18]. However, most of these applications, if not all, require lower thermal expansion than typically presented by neat Al and Al alloys. In Al-Si alloys, the CTE value is generally controlled by silicon content since its increment proportionally reduces linear CTE. Oddone et al. [13] reported that the high silicon content (~14 wt.%) in Alumix-231 reduces the linear CTE from around 23 × 10^−6^ °C^−1^ to approximately 18.5 × 10^−6^ °C^−1^, as generally reported for neat aluminum.

In an attempt to increase the portfolio of Alumix-231, stiff ceramics have been added to reinforce the alloy, principally to raise its mechanical properties (hardness, tensile, and wear resistance). Callioglu et al. [19] investigated the effects of cold uniaxial pressing (600 MPa) and hot extrusion (565 °C, via P/M) on microstructures and mechanical properties (hardness and tensile strength) of Alumix-231 based MMC, reinforced with SiC and B_4_C particles (5, 10 and 20 wt.%). It was concluded that the addition of SiC and B_4_C, together with the extrusion process, were beneficial to densification (<1% porosity) and the hardness of composites. For example, the composite reinforced with 20 wt.% SiC presented the highest hardness—close to 150 HV5.

In the research conducted by Rudianto et al. [20], the sintering conditions of A359 (Al-9Si-0.5Mg-0.2Cu-0.2Fe-0.2Ti) with 20 wt.% of SiC were studied, and the effect of the addition of 25%, 50%, and 75 wt.% of Alumix-231—replacing partially for A359—on mechanical properties was additionally evaluated. The samples were compacted at 700 MPa and the green bodies sintered at 560 °C for 1 h. MMC with the addition of 75 wt.% of Alumix-231 had the highest relative green density (92%) and also presented the highest density (96.8%) after sintering. The response to the hardness test of this composite was close to 100 HRB in the T1 condition, and to 50 HRD in the aged condition (T6)—higher than in other MMCs.

Bang et al. [21] developed an MMC based on Alumix-231, reinforced with the composite powder made from Al-9Si/20 vol% of SiC through the P/M processing route. The authors investigated the microstructure and mechanical properties of the MMC specimens when sintered at 580 °C/1 h, and found a high density (~99%), high tensile strength (230 MPa), and a 5.24% increase in elongation.

Fused silica is a low-cost amorphous ceramic, characterized by near-zero thermal expansion (0.54 × 10^−6^ C^−1^, 0–800 °C) [22], low thermal conductivity, low density (~2.25 g cm^−3^), and a high thermal shock resistance [23]. However, the relatively low mechanical strength of this ceramic is insufficient to meet some requirements, especially for application in hypersonic spacecraft [22]. Fused silica is also characterized by relatively low Young’s modulus (72 GPa) [24]. Although fused silica has some limitations, concerning mechanical properties, it has been used to reinforce Al-Si based alloys and has given good results in terms of hardness increase and uniform microstructures [24,25]. The study conducted by Magesh et al. [24] reported the development of MMCs based on the aluminum alloy LM 13 (Al-12Si), reinforced with 9, 12, and 15 wt.% fused silica, using the casting method. As a consequence, an increase in hardness from 86.2 HV (matrix) to 97.7 HV (15 wt.% of fused silica) was measured.

Hemanth [25] used A356 Al-alloy (Al-7Si-0.4Mg-0.2Cu-0.2Fe-0.1Zn-0.1Mn) and 3, 6, 9, and 12 wt.% of fused silica to develop an MMC by casting matrix material. Fused silica particles, preheated to 500 °C, were evenly introduced into the molten alloy. The microstructure, hardness, and wear behavior of the resulting cast composites were studied and an increase of hardness was confirmed with the addition of fused silica in comparison to neat alloy.

However, neither Magesh et al. [24] nor Hemanth [25] investigated the effect of fused silica on CTE of the studied Al-Si based matrices.

Therefore, the main goal of this study was to prepare, through P/M, T1 pellets of MMCs based on Alumix-231 with the addition of different volume percentages of fused silica (5, 10, 15 and 20 vol.%) to reduce its linear CTE, without compromising low density and hardness level, inherent to Alumix-231.

## 2. Experimental

### 2.1. Materials

Alumix-231 powder was acquired from Kymera International (Velden, Germany). The powder particles are irregularly shaped with sizes of 90 µm (D50). In accordance with the manufacturer, the theoretical density of the alloy is 2.677 g cm^−3^ with a melting point of 570 °C. The nominal chemical composition of Alumix-231 is presented in Table 1.

Fused silica powder was acquired from Dupré Minerals (Newcastle, United Kingdom), with purity >99.8%. The powder particles are polygonal in shape with sizes of 14–18 μm (D50). In accordance with the manufacturer, the theoretical density of fused silica is 2.250 g cm^−3^ and its melting point is above 1094 °C.

### 2.2. Mixing of Alumix-231 and Fused Silica Powders

The powders were firstly weighted at a BEL Engineering M214Ai digital analytical balance (with a resolution of 0.0001 g and a maximum capacity of 210 g).

Then, Alumix-231 and fused silica powders were pre-mixed manually, for 5 min, in an agate mortar. The pre-mixtures were then placed in a zirconia vessel with a capacity of 5–15 g. Two zirconia balls—each with a diameter of 10 mm and a mass of 6.04 g—were added, keeping the ball to powder ratio 2:1. Ball milling was carried out in a SPEX 8000M Mixer/Mill ball mill for 20 min, at a rotation speed of 1425 rpm.

### 2.3. Preparation of Green and Sintered MMC Pellets

The as-mixed powders, identified as Alumix-231/5, 10, 15, or 20 vol.% fused silica, were uniaxially pressed at 700 MPa, using a MARCON MPH-60 Uniaxial Hydraulic Press (60 Ton), for 2 min, in pellets of 16 mm in diameter and 4 mm of height.

The as-prepared green pellets were further sintered in a Fortelab Tubular Oven to obtain metal matrix composites (MMCs). Firstly, green pellets were preheated to 410 °C for 30 min for delubrication, with a pressure of 1.2 mbar and a flow of 2 L min^−1^ of N_2_. The heating rate of 10 °C min^−1^ was applied. Secondly, the samples were heated at the same rate, and maintained at 565, 570, or 575 °C for 90 min in N_2_ atmosphere to prepare sintered T1 pellets. Afterward, the pellets were cooled inside the oven in a controlled fashion to room temperature.

### 2.4. Characterization of Green and Sintered MMC Pellets

Differential Scanning Calorimetry (DSC) analyses were performed on 12 mg of green body fragments. The DSC analysis was performed in triplicate to guarantee the reproducibility of the tests, using a Simultaneous Thermal Analyzer (STA 6000, Perkin-Elmer, Thane, India) with a flow of 20 mL min^−1^ of N_2_ in the temperature range between 25 and 800 °C, under a heating rate of 10 °C min^−1^.

X-ray powder diffraction (XRPD) was performed using a Bruker D8-Advance X-ray diffractometer with a voltage of 40.0 KV and a current of 30.0 mA, in the range between 20 and 45° (2θ), with the acquisition time of 5 s per step of 0.02°. The powder samples for XRPD of neat Alumix-231 and MMCs with different volume percentages of fused silica (5, 10, 15, and 20 vol.%) were obtained by the crushing of pellets with the aid of agate pestle, and milled manually in an agate mortar until fine powders were formed. The as-prepared samples were then mounted on a zero-background sample holder.

The microstructure of sintered neat Alumix-231 and MMCs were analyzed on carbon coated samples by Scanning Electronic Microscopy (SEM), using a Hitachi TM3000 equipment (Hitachi High-Technologies, Tokyo, Japan), operating in back-scatter electron mode. The samples dedicated to SEM were sanded, using a polisher with sandpapers (220, 400, 600, and 1000) for 3 min, and polished with a diamond paste of 9 μm, 6 μm, 1 μm, and 1/4 μm for 2 min.

The green and sintered densities of the pellets were performed in triplicate and determined by Archimedes’ Principle, using a BEL Engineering M214Ai digital analytical balance, according to ASTM B962-13 [26].

Rockwell hardness was measured using a Pantec RASN-RS Durometer on both the “B” and “E” scales, using a 1/16” hardened steel ball penetrator with an applied load of 100 Kgf. Five measurements were made on each sintered sample of MMCs to obtain the mean value and the standard deviation.

Vickers microhardness was measured using a Shimadzu HMV-2000, with a square base diamond pyramid indenter with an angle of 136° between the faces, and with indentation time of 15 s, using a load of 2.94 N. Fifteen measurements were made on each sample, neat Alumix-231, and MMCs sintered at 565 °C.

Dilatometry of the sintered, neat Alumix-231, and MMC quadrangular prismatic bodies (8 mm × 4 mm × 4 mm), cut using Buehler Isomet 100 with a diamond blade, was performed in triplicate using a Netzsch Dil 402C Pushrod dilatometer. First, the equipment was calibrated with an alumina standard, provided by Netzsch, with the same geometry as the sintered pellets. The heating occurred at a rate of 10 °C min^−1^, in an air atmosphere between 25 and 500 °C, followed by cooling at the same rate to room temperature. CTEs of the sintered MMC bodies were calculated between room temperature and 400 °C, according to ASTM E228-11 [27]. Standard deviations for the as-obtained CTEs were calculated based on three different measurements, coming from three different prismatic bodies of the same specimen (neat Alumix-231 or MMC).

## 3. Results and Discussion

### 3.1. DSC Characterization of Alumix-231 and MMCs Green Pellets

**Figure 1:**
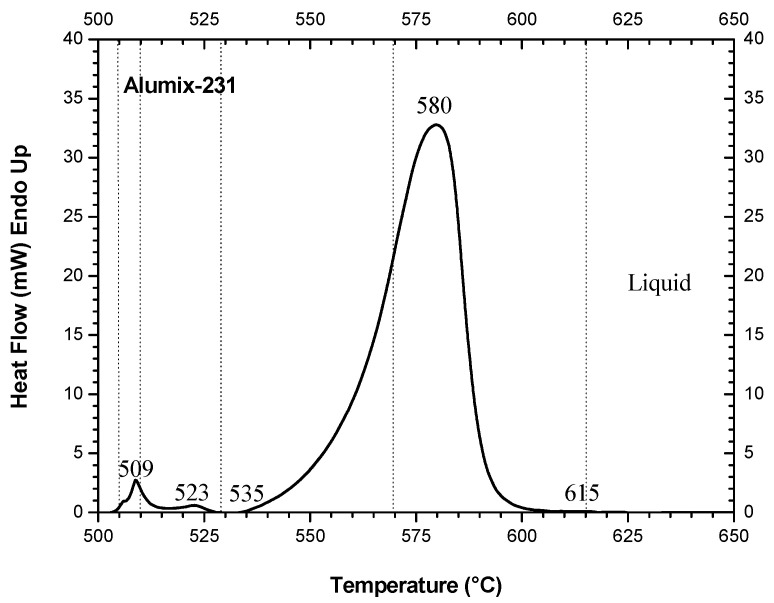
DSC curve of the green pellet of neat Alumix-231.

Figure 1 shows the DSC trace of the green body of neat Alumix-231, evidencing the presence of three endothermic features with the maxima situated at 509, 523, and 580 °C.

Arribas et al. [15] suggested that the two lower intensity endothermic events, located at 509 °C and 523 °C, respectively (Figure 1), were associated with the melting of the intermetallic compounds, such as θ-CuAl_2_ and the Q phase (Cu_2_Mg_8_Si_6_Al_5_) [21]. In addition, according to Arribas et al. [15], the most intense and broad endothermic peak, with the onset at ~535 °C (Figure 1), is an indication of the beginning of eutectic melting of Al solid solution and Si and, concomitantly, represents the onset of liquid phase sintering. In accordance with these authors, the silicon particles continued to melt until ∼615 °C, above which the Alumix-231 is completely liquid [15], confirmed by our findings (Figure 1).

**Figure 2:**
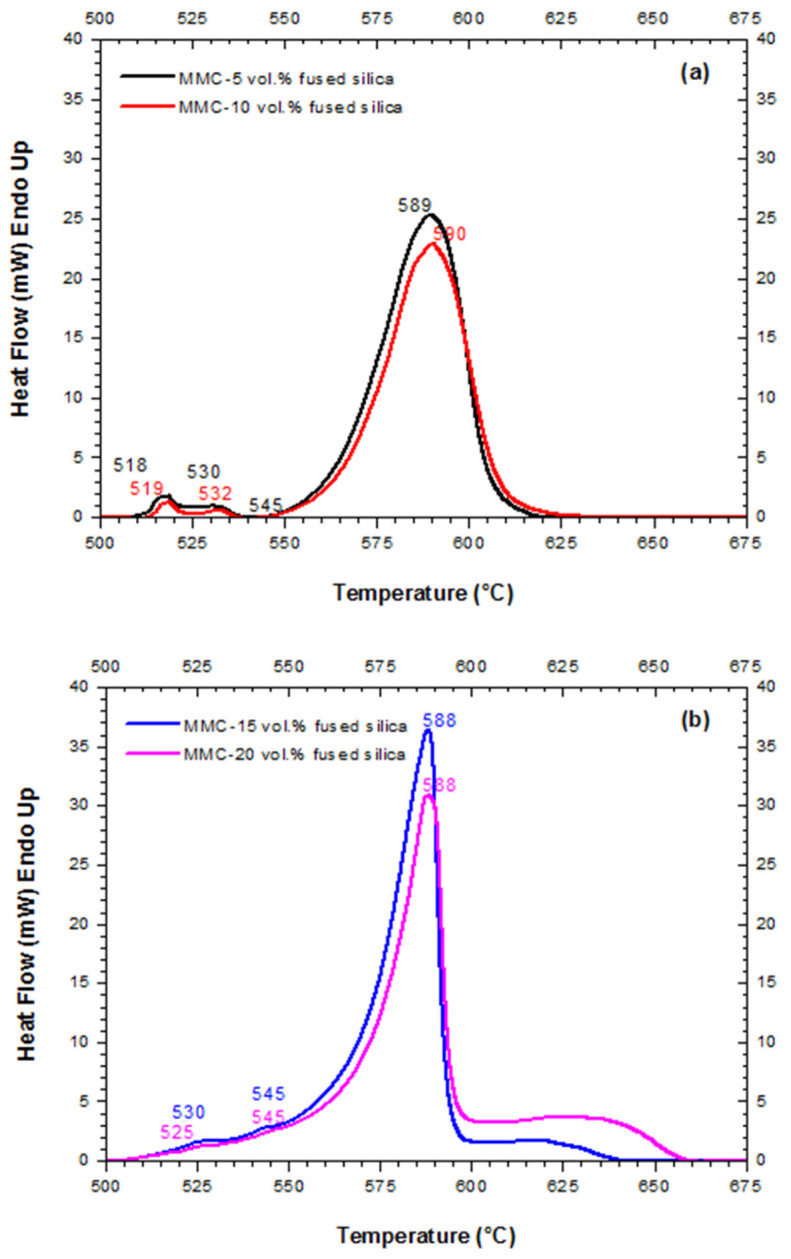
DSC curves of the green bodies of (**a**) MMC-5 and 10 vol.% of fused silica and (**b**) MMC-15 and 20 vol.% of fused silica.

Figure 2a,b show the DSC curves of MMC green bodies with 5 and 10 vol.%, and with 15 and 20 vol.% of fused silica, respectively.

Endothermic peaks of MMC-5 and 10 vol.% (Figure 2a) are very similar to each other and to the DSC curve of neat Alumix-231 (Figure 1). On the other hand, the first two endothermic peaks observed in Figure 2b were almost imperceptible for the MMC-15 and 20 vol.%. The most intense endothermic peak for MMCs-5 and 10 vol.% (Figure 2a) and for MMCs-15 and 20 vol.% (Figure 2b), situated, respectively, at 589 and 590 °C, and 588 °C, underwent a minor change concerning the endothermic peak of the neat matrix, located at 580 °C (Figure 1). On the other hand, the temperature extension of the main melting event to the higher temperatures, especially for MMC-20 vol.% (approaching 660 °C), is possibly due to the effect of fused silica on the melting of silicon crystals.

### 3.2. Effects of Compaction Pressure on the Green Density of Alumix-231 and MMCs

**Figure 3:**
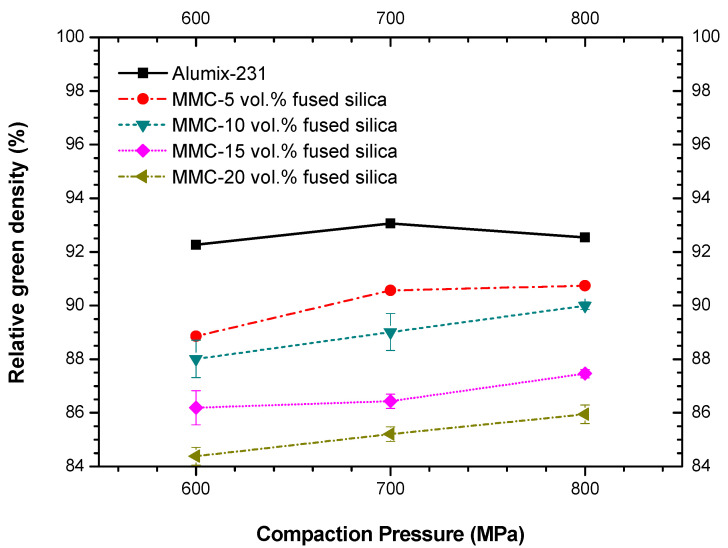
Green densities for neat Alumix-231 and MMCs as a function of uniaxial compaction pressure. For some samples error bars are smaller than the symbols.

Heard et al. [12] demonstrated that the ideal compaction pressure of neat Alumix-231 was 600 MPa, with a green density as high as ~92% of the theoretical one. To optimize the compaction pressure for the preparation of highly dense green bodies of MMCs (Alumix-231/fused silica), uniaxial pressing was carried out at different pressures, such as 600, 700, and 800 MPa. Figure 3 presents the variation of green densities as a function of compaction pressure for neat Alumix-231 and MMCs with different amounts of fused silica.

Green densities of MMCs were lower than for neat Alumix-231, due to lower compressibility of ceramic fraction, and slightly increased with increasing compaction pressure. Higher amounts of fused silica caused a decrease of green density in comparison to neat alloy, however, the reduction in green density was lower than 10% even for the MMC-20 vol.%. It is generally not practical—or recommended—to compact at a pressure as high as 800 MPa, with regard to safety issues, and excessive and accelerated wear of equipment tooling. In addition, the gain in density when 800 MPa was applied was small in comparison to 700 MPa. As such, 700 MPa was considered as the optimized pressure for compaction of MMCs, since it combined high density with safety requirements.

### 3.3. Effects of Fused Silica Addition and Sintering Temperatures on Thermal Expansion of MMCs

**Figure 4:**
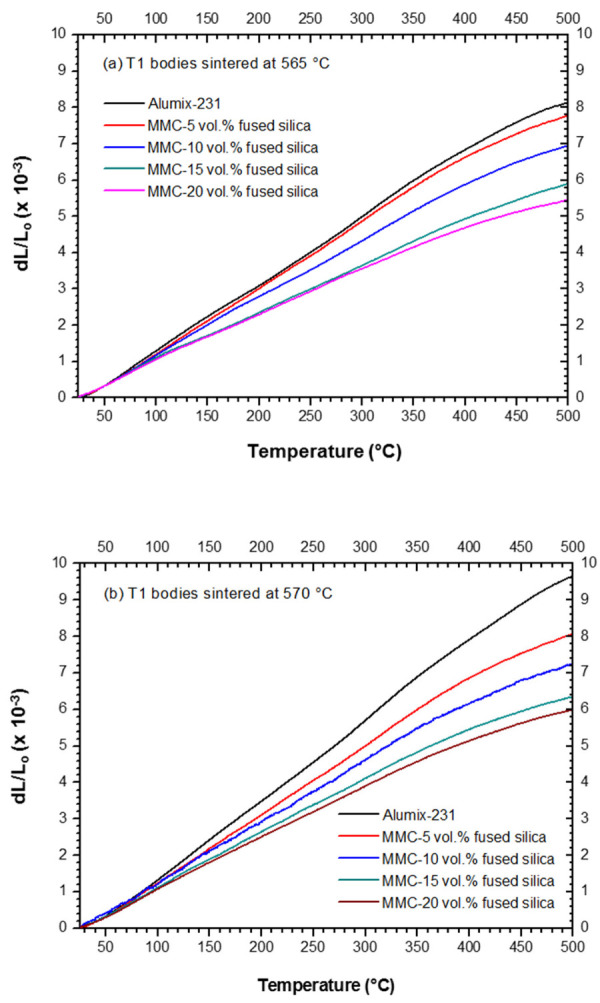

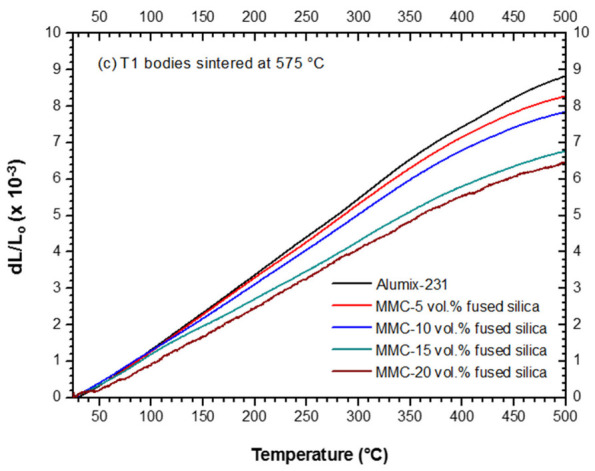
Thermal expansion of neat Alumix-231 and MMCs (**a**) sintered at 565 °C, (**b**) 570 °C, and (**c**) 575 °C.

Figure 4a–c shows thermal expansions (i.e., thermal deformation) of neat Alumix-231 and MMCs, sintered at 565, 570, and 575 °C, respectively.

**Figure 5:**
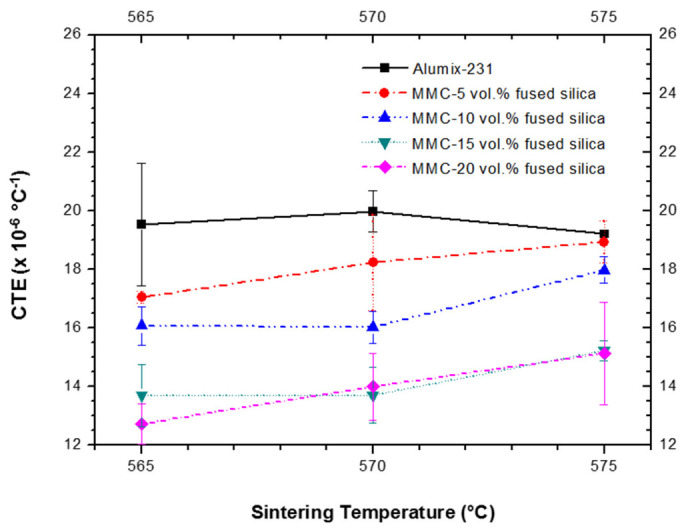
CTEs of neat Alumix-231 and MMCs *versus* sintering temperatures.

A strong effect of fused silica on the reduction of thermal expansion of the MMCs in respect to the thermal expansion of neat Alumix-231 was observed (Figure 4) and quantified for the technologically important temperature range, between 25 and 400 °C (Figure 5). It is worth noting that at temperatures approximately higher than 400 °C, a reduction of CTEs in neat alloy as well as in MMCs was evident in respect to the lower temperatures (Figure 4), verified through reduction of the slopes of the dilatometric curves. This peculiar feature of CTE was previously reported and explained by Hahn and Amstrong [28] for Al-Si eutectic alloys, and subsequently confirmed for other Al-Si alloys, such as AlSi20 [29]. The reduction of CTE in Al-Si alloys at the temperatures approximately >400 °C, and, therefore, in Alumix-231 and MMCs, is owing to an increment of solubility of Si inside Al face-centered cubic structure with the increase of temperature, causing shrinkage of the unit-cell as Al is becoming richer in Si substitutional solute. Therefore, this phenomenon partially counteracts positive thermal expansion caused by anharmonicity of the lattice and, in accordance to Hahn and Amstrong [28], could be as high as −2.2 × 10^−6^ °C^−1^ at 450 °C.

Figure 5 presents a comparison between the CTEs of the neat Alumix-231 and the MMCs. A significant reduction of CTEs of MMCs in comparison to the CTE of neat Alumix-231 alloy is evidenced. This is especially evident for the MMCs with 15 and 20 vol.% of fused silica, sintered at 565 °C.

As shown in Figure 5, the addition of 5 and 10 vol.% of fused silica provided, respectively, a reduction in CTE of ~12.7% and ~17.7% for the sintering temperature of 565 °C, in respect to the CTE of neat Alumix-231. In addition, the MMCs with 15 and 20 vol.% of fused silica, sintered at 565 °C, exhibited CTEs of 13.70 and 12.73 × 10^−6^ °C^−1^, respectively, and a reduction of CTEs as high as 29.9% and 34.8%. The decrease of CTE in our MMCs is in accordance to that which is generally observed for this class of materials with the increase of ceramic phase volume [30,31], and the reached values of CTE are rather low for an Al-Si based MMC. This is especially true for the MMCs with 15 and 20 vol.% of fused silica, and the CTE values are consistent with the desired CTE for automotive applications, which consider substitution of heavy cast iron components.

With the increase of the sintering temperature from 565 to 575 °C, a slight increment in the CTEs of the MMCs was observed. It should be noticed that although the increment of CTE with sintering temperatures is verified for all four MMCs, the values are still within the standard deviation. However, a possible explanation for this feature of CTE might be related to a higher content of intermetallic θ-CuAl_2_ in the pellets sintered at 565 °C compared with the MMCs sintered at higher temperatures, as confirmed by XRPD (Supplementary Information, Figure S1), since this intermetallic phase has lower CTE than neat Al [32]. Alumix-231 is already a composite containing two intermetallic phases and silicon embedded in Al-matrix. Therefore, the absence of two stiffer intermetallic phases with lower CTEs at higher sintering temperatures (Figure S1) would affect the overall CTE of MMCs and increase it.

To better understand the effect of the residual stress on the *CTE*, owing to the mismatch between Alumix-231 and fused silica, the thermal expansion mismatch residual stress, *P_CTE_*, generated inside a composite, was calculated from the Equation (1) [33]:

(1) $$P_{CTE}=\Delta \alpha_{l}\Delta T{\left[\frac{1+v_{m}}{2Y_{m}}+\frac{1-2v_{f}}{Y_{f}}\right]}^{-1}\;$$

where Δ*α_ℓ_* is the difference between the linear CTE of the matrix *α_ℓ_*(m) (Figure 5) and that of the ceramic reinforcement *α_l_*(*f*) = 0.54 × 10^−6^ °C^−1^ [22], and Δ*T* is the difference between the processing and room temperatures, while *Y_m_* = 83 GPa [12], *Y_f_* = 72 GPa [24], *ʋ_m_* = 0.33 and *ʋ_f_* = 0.17 are Young’s moduli and Poisson coefficients of the matrix and filler, respectively.

**Figure 6:**
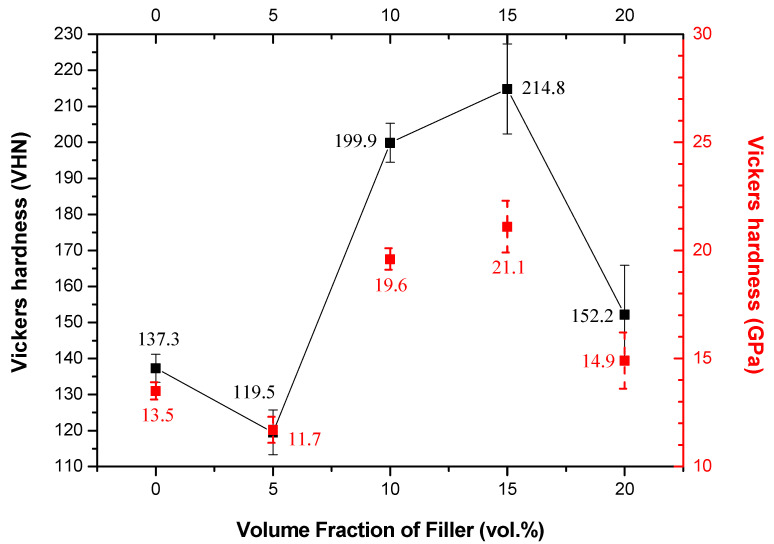
Vickers hardness of neat Alumix-231 and MMCs sintered at 565 °C *versus* volume fraction of filler.

Therefore, residual stress of Alumix-231/fused silica MMCs, sintered at 565 °C, was calculated to be as high as 597 MPa. This stress far exceeded the yield stress of the Alumix-231 matrix (∼210 MPa [34]) and, therefore, it can be deduced that the matrix should be plastically deformed at room temperature. Indeed, Figure 6 confirms a significant hardening of the Alumix-231 matrix within MMCs for a majority of MMCs, except for MMC with 5% of fused silica, a consequence of dislocation formation and their entanglements, caused by residual stress.

**Figure 7:**
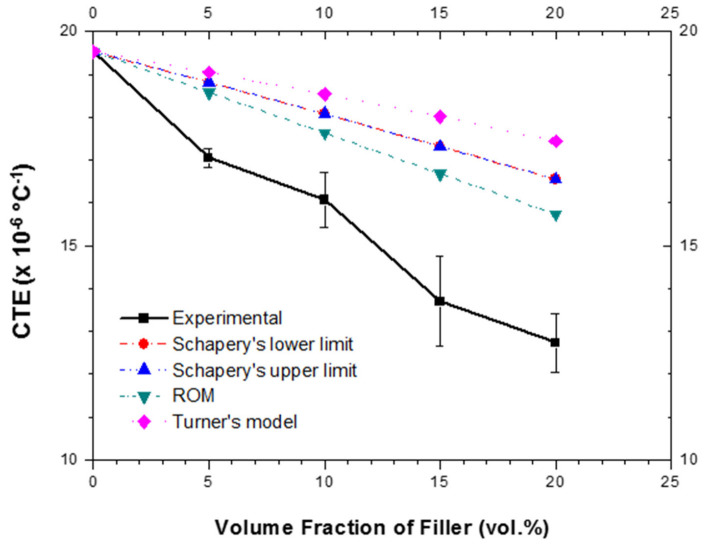
Experimental CTEs (between 25 and 400 °C) of MMC (Alumix-231/fused silica) sintered at 565 °C and CTEs predicted by different micromechanical models.

Micromechanical models, such as Rule of Mixture, Turner’s, and Schapery’s, have been applied to predict CTEs of our MMCs sintered at 565 °C (the most promising temperature in our study) as a function of volume fraction of fused silica particles (Figure 7). Materials properties used for calculation of micromechanical models are listed in Supplementary Information, Table S1. Interestingly, the measured CTEs were lower than the calculated ones by the proposed models. This feature has been previously reported for Al/SiC MMCs and was ascribed to hardening of the matrix by residual stress and the fact that the considered micromechanical models do not take strain hardening into account [35]. Considering the significant hardening inside the metal matrix (Figure 6), it seems that the discrepancy between calculated and measured values of CTEs in our MMCs might have the same origin as in the Al/SiC.

An efficiency factor has been proposed by Yang et al. [36] to evaluate the efficiency of a ceramic filler for the reduction of CTE inside MMC. In our study, however, we used a modified efficiency factor (*R*), Equation (2), in line with the one used for evaluation of the strengthening effect of ceramic fillers on the yield strength of MMCs [37,38]:

(2) $$R=\left|\frac{\alpha_{c}-\alpha_{m}}{V_{p}\;\alpha_{m}}\right|\;$$

where *α_c_* is the coefficient of thermal expansion in MMCs, *α_m_* is coefficient of thermal expansion in a metallic matrix, and *V_p_* is the volume fraction of the ceramic phase inside MMCs.

**Figure 8:**
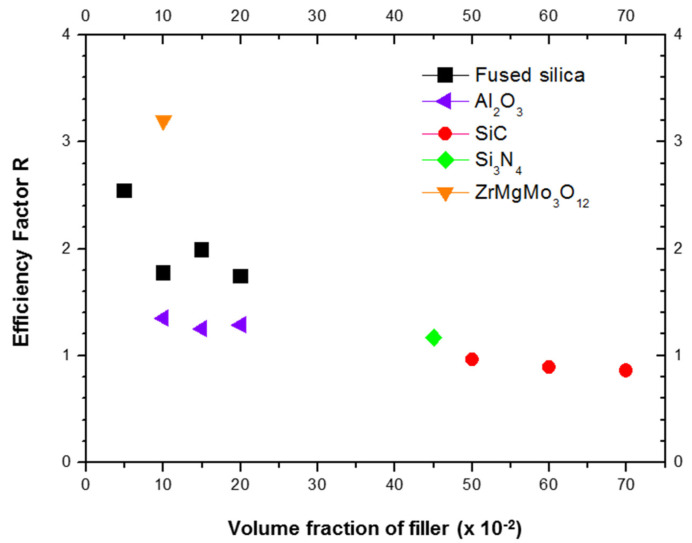
Efficiency factors of ceramic fillers on CTE reduction *versus* ceramic volume fractions. Efficiency factors of fused silica fillers within Alumix-231 matrix, sintered at 565 °C, were plotted. Efficiency factors for ZrMgMo_3_O_12_, SiC, Al_2_O_3_ and Si_3_N_4_ are available from literature [36,39,40,41].

Figure 8 illustrates very high efficiency factors for all tested volume fractions of fused silica within the Alumix-231 matrix. The values of fused silica efficiency factors are significantly higher than those reported for some other traditional ceramics such as Al_2_O_3_ [39], SiC [40], and Si_3_N_4_ [41]. Only a new near-zero thermal expansion phase, such as ZrMgMo_3_O_12_ [36], embedded in a neat Al matrix, presented a superior value of efficiency factor in comparison to fused silica.

### 3.4. Effects of Fused Silica Addition and Sintering Temperature on Density and Hardness

Heard et al. [12] optimized the sintering conditions of Alumix-231 (560 °C/60 min), achieving a relative density of 98% and a Rockwell hardness of ~87.0 HRE. In our study, samples of MMCs were sintered at 565, 570, and 575 °C, for 90 min.

**Figure 9:**
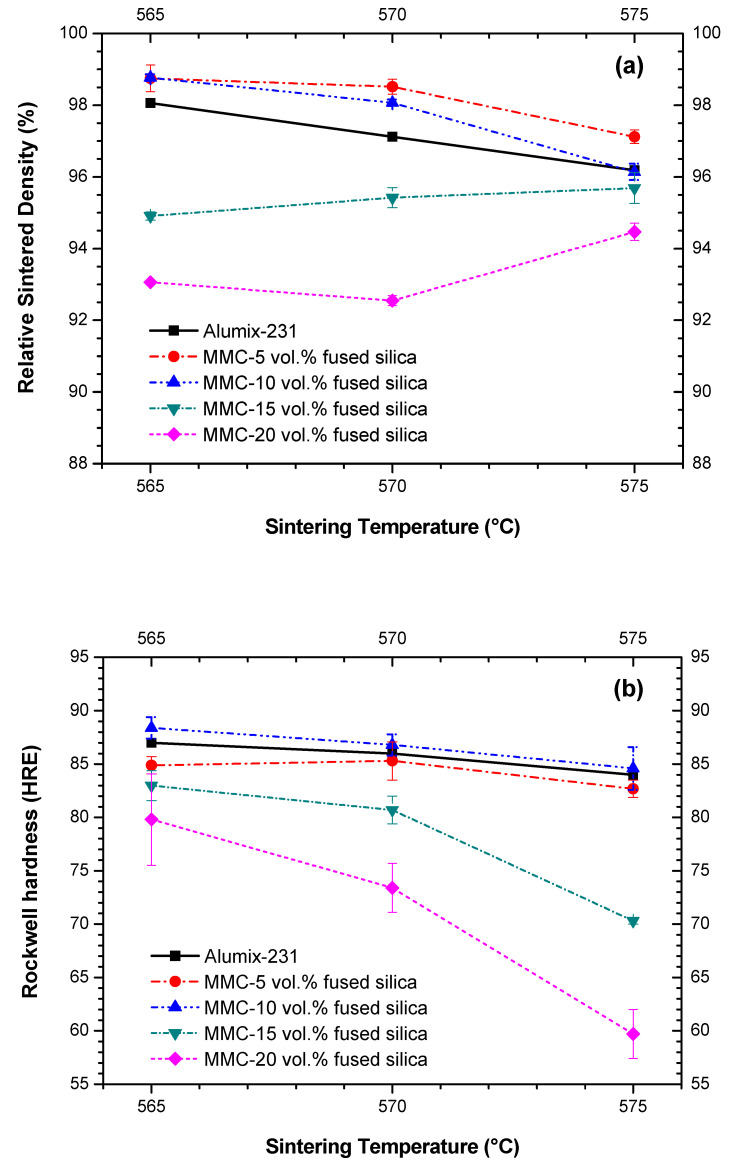
Variation of density and hardness of MMCs, in respect to sintering temperature, (**a**) relative sintered density, and (**b**) Rockwell hardness, *versus* sintering temperatures. Densities and hardness of neat Alumix-231 sintered at 565, 570, and 575 °C and measured in our samples were essentially the same as those measured by Heard et al. [12].

Figure 9a,b show, respectively, the variation of relative density and Rockwell hardness (E scale) of MMCs reinforced with 5, 10, 15, and 20 vol.% of fused silica in respect to sintering temperatures, and in comparison to the density and hardness values previously measured for Alumix-231 [12]. For the most promising sintering temperature of 565 °C, the MMC with 5 and 10 vol.% of fused silica showed slightly higher densities (~1%) than for neat Alumix-231 (Figure 9a). However, further increase of fused silica content to 15 and 20 vol.% reduced relative density from higher than 98% of theoretical one to 95% and 93%, respectively. This reduction is relatively low—not higher than 6%—and is a consequence of the closed voids formed due to fused silica particles agglomeration, which prevents these regions from liquid phase sintering [42]. Higher sintering temperatures, such as 575 °C, apparently approximated density values of MMCs and neat Alumix-231. The increase of the densities in the MMC with 15 and 20 vol.% of fused silica at 575 °C seems to be a consequence of increasing liquid content as the temperature of sintering increased, in accordance to DSC curves (Figure 2), permitting a more efficient filling of the agglomeration voids. On the other hand, the decrease of the densities in the MMC with lower fused silica contents (5 and 10 vol.%), also previously observed for the neat Alumix-231 [12], can be rationalized in terms of overabundance of liquid-phase as suggested by Heard et al. [12]. Nevertheless, the sintering temperature of 565 °C has priority over other temperatures since contributes to the lowest CTEs.

Regarding the hardness of the MMCs, there are several aspects—evidenced in Figure 9b—to be discussed. In addition, special attention should be given to the MMCs sintered at 565 °C, since they presented the lowest CTEs when compared to the MMCs sintered at higher temperatures, and therefore attended to one of the major goals of this study. Furthermore, fused silica particles inside these MMCs showed one of the highest efficiency factors among other ceramic fillers, as previously demonstrated (Figure 8).

It may be expected that the addition of fused silica would slightly decrease hardness since Young’s modulus of fused silica is lower than for Alumix-231 (72 and 83 GPa, respectively). Smaller amounts of fused silica (5 and 10 vol.%) maintained a hardness very close to that measured for Alumix-231 [12]. However, lower rigidity of fused silica might be one of the origins of hardness reduction for MMCs with 15 and 20 vol.%, not higher than 3 and 5% when sintered at 565 °C, in comparison to neat Alumix-231. Another, even more significant reason for the reduction of hardness for this set of MMCs (15 and 20 vol.%) is the reduction of density (and consequent increase of porosity), as previously observed (Figure 9a)—a feature that was not verified for the composites with 5 and 10 vol.% of fused silica.

**Figure 10:**
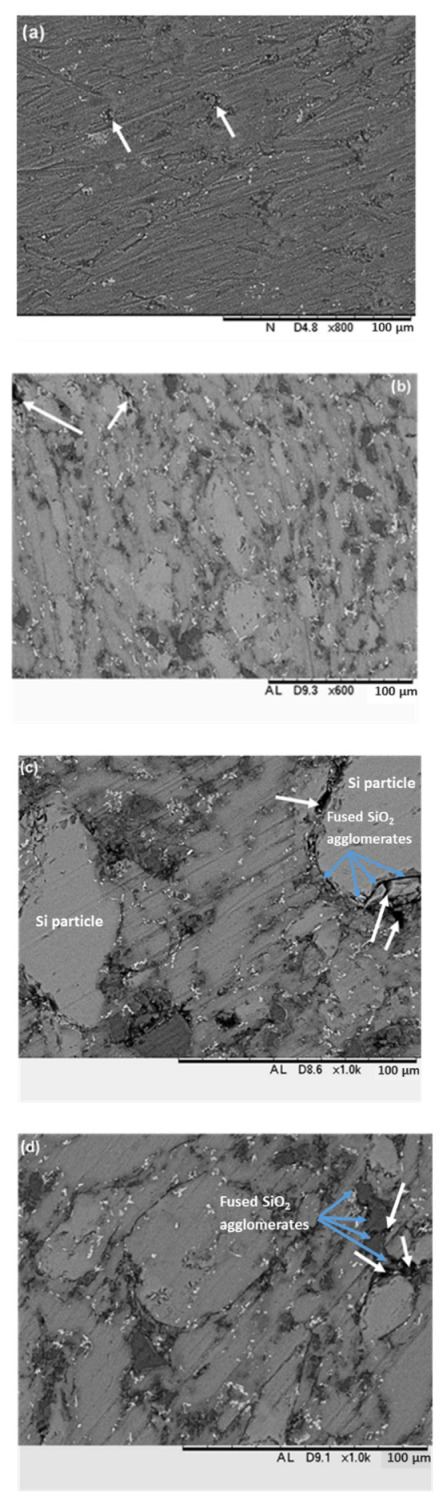
Porosity in (**a**) neat Alumix-231, (**b**) MMC-10 vol.%, (**c**) MMC-15 vol.% and (**d**) MMC-20 vol.%. White arrows show pores; blue arrows point to fused silica agglomerates.

An increase of porosity with the increase of fused silica content was verified, in addition, through SEM analysis (Figure 10). Porosity in neat Alumix-231 and in MMCs with fused silica content ≤ 10 vol.% is low and pores are smaller in size (Figure 10a,b). However, with the increase of fused silica content to 15 and 20 vol.%, porosity increased in percentage and pores in size and were generally associated with agglomerates of fused silica particles (Figure 10c,d). Another source of pores could be the interfaces between Si particles and the neat Al matrix (Figure 10c), probably due to residual stress between these two phases of Alumix-231.

It is worth noting that the hardening of the Alumix-231 matrix, as documented in Figure 6, did not lead to an increase in the overall hardness of MMC.

Although MMCs obtained at 570 and 575 °C are not that promising in terms of CTE, it is worth mentioning that the hardness of those MMCs with 15 and 20 vol.% of fused silica decreased substantially for 570 °C, and particularly for the sintering temperature of 575 °C. A similar decrease of mechanical properties was reported for neat Alumix-231 at temperatures higher than 560 °C and was ascribed to the coarsening of the matrix microstructure [12]. In addition, a similar hardness reduction was observed for ZrMgMo_3_O_12_/2024Al composite for the highest sintering temperature and was ascribed to grain coarsening, in accordance with the Hall-Petch relationship [36]. As such, this mechanism might be also responsible for the reduction of mechanical properties for the MMCs sintered at 570 and 575 °C. Furthermore, the reduction of hardness at the temperature higher than 565 °C might be partially caused by the disappearance of the hard intermetallic θ-CuAl_2_ phase, as documented by XRPD (Supplementary Information, Figure S1).

## 4. Conclusions

This study found Alumix-231 based composites sintered at 565 °C, with the addition of 15 and 20 vol.% of fused silica and developed through P/M with the aid of liquid phase, can reach CTEs as low as 13.70 and 12.73 × 10^−6^ °C^−1^ (between 25 and 400 °C), respectively. These CTEs values are attractive for light MMCs to be used as a substitution of heavy cast iron components for automotive applications. In addition, fused silica showed to be a very efficient traditional and low-cost ceramic filler, in comparison to Al_2_O_3_, SiC, and Si_3_N_4_, for the reduction of CTE of Al-based alloys.

The measured values of CTEs (between 25 and 400 °C) for the Alumix-231 based MMCs (5 to 20 vol.% of fused silica) at 565 °C are lower than those predicted by several micromechanical models, possibly due to the strain hardening of the matrix, an effect not considered in these models.

Importantly, Alumix-231 based MMCs with the addition of 15 and 20 vol.% of fused silica exhibited only a slight decrease in density and Rockwell hardness in comparison to neat alloy—as low as 6% and 5%, respectively. As such, the developed Alumix-231 based MMC, with the addition of 15 and 20 vol.% of fused silica did not have their density and hardness compromised in comparison to the neat matrix, while showing a strong reduction in CTEs (29.9% and 34.8%, respectively).

Future thorough electron microscopy studies should further contribute to a full understanding of mechanisms that are responsible for a slight decrease of density and hardness in Alumix-231 based MMCs sintered at 565 °C, with the addition of 15 and 20 vol.% of fused silica.

## Figures and Tables

**Table 1:** Nominal chemical composition of Alumix-231, in accordance to the manufacturer.

Chemical Element	Al	Si	Cu	Mg	Licowax C P
**wt.%**	80.16	15.10	2.80	0.50	1.44

## Data Availability

The data underlying this article will be shared on reasonable requestfrom the corresponding author.

## Supplementary Materials

The following supporting information can be downloaded at: https://www.mdpi.com/article/10.3390/ma15103476/s1, Figure S1: XRPD patterns of MMC-5 vol.%, sintered at 565 and 575 °C, Table S1: Thermomechanical properties of Alumix-231 and fused silica.
