# Supplementary material for: Effects of Fused Silica Addition on Thermal Expansion, Density, and Hardness of Alumix-231 Based Composites
Source: Materials (Basel). 2022 May 12;15(10):3476. doi: 10.3390/ma15103476 (PMC9143313; doi:10.3390/ma15103476)
Supplement: Supplementary file 1 [file materials-15-03476-s001.zip › materials-1701004-supplementary.pdf]

Supplementary Material

EFFECTS OF FUSED SILICA ADDITION ON THERMAL EXPANSION, DENSITY AND HARDNESS OF ALUMIX-231 BASED METAL MATRIX COMPOSITES

L.M. Rodrigues, B.A. Marinkovic*

Department of Chemical and Materials Engineering, Pontifícia Universidade Católica do Rio de Janeiro (PUC-Rio), Rio de Janeiro, RJ, Brazil

*e-mail: bojan@puc-rio.br

XRPD of pellets MMC-5 vol.% of fused silica, sintered at 565 and 575 °C.

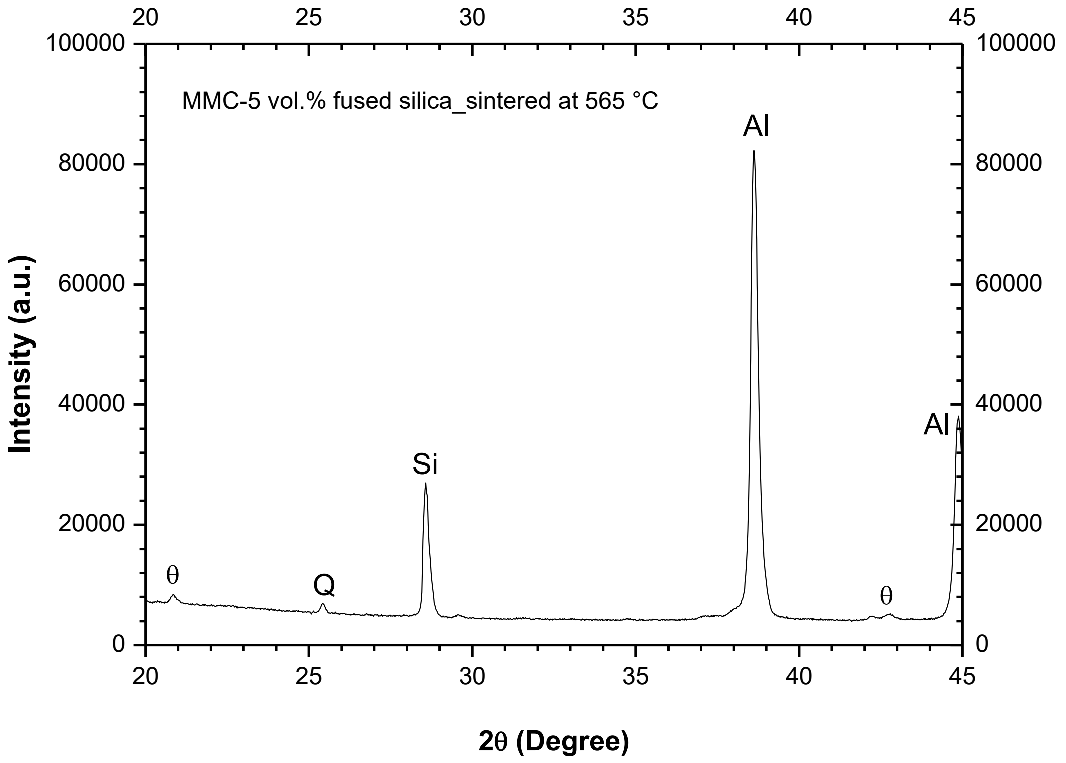

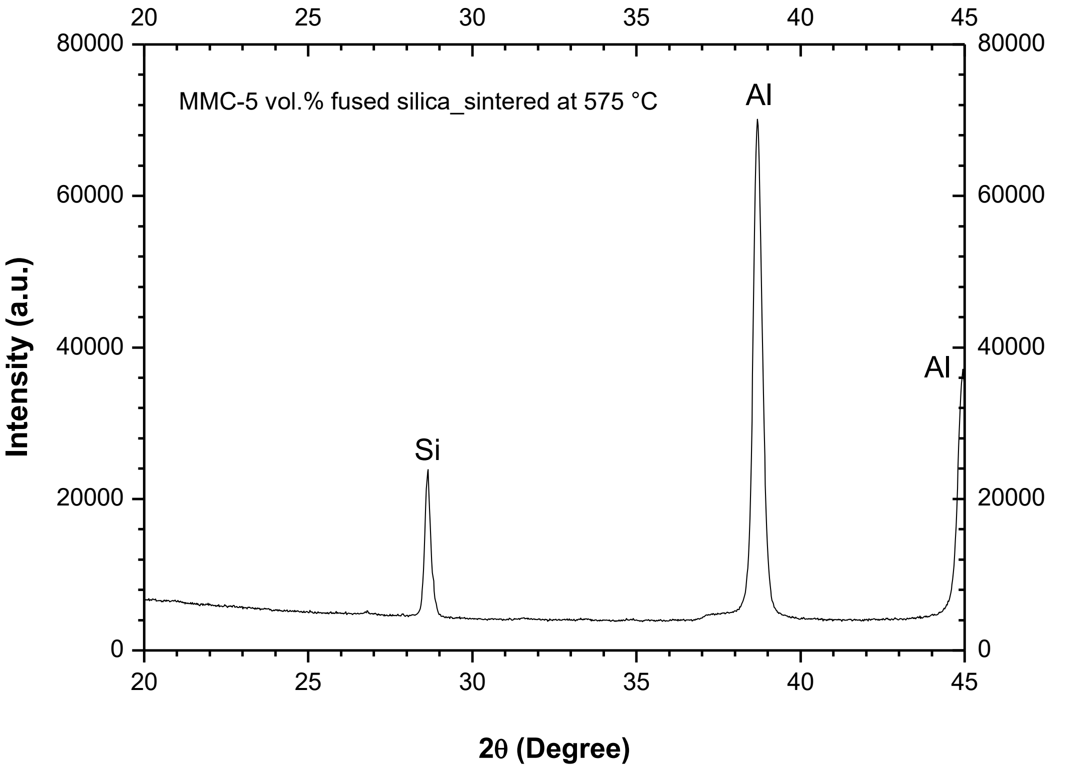

Figure S1: XRPD patterns of MMC-5 vol.%, sintered at 565 and 575 °C.

Thermomechanical properties.

Table S1: Thermomechanical properties of Alumix-231 and fused silica.

**Material**	**Young modulus (GPa)**	**Shear modulus (GPa)**	**Bulk modulus (GPa)**	**Poisson coefficient**	**Coefficient of thermal expansion ($x\ 10^{-6}\ C^{-1}$)**	**Density (g/cm^3)**
**Alumix-231**	83 [12]	31.13[a]	83.00[b]	0.33	18.50 [13]	2.668 [20] / 2.634 [12]
**Fused silica**	72 [24]	31.00[a]	41.00[b]	0.17	0.54 [22]	2.250 [23]

a,b—calculated using the relationships between elastic constants.
